# Supplementary figures and images for: ER-to-lysosome Ca2+ refilling followed by K+ efflux-coupled store-operated Ca2+ entry in inflammasome activation and metabolic inflammation
Source: eLife. 2024 Jul 2;12:RP87561. doi: 10.7554/eLife.87561 (PMC11219040; doi:10.7554/eLife.87561)

**Figure. 1E**

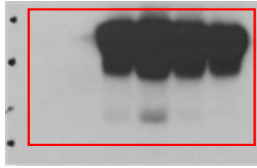

**IL-1 $\beta$**

Supplement: Figure 1—source data 2. [file elife-87561-fig1-data2.pdf]

**Figure. 2C**

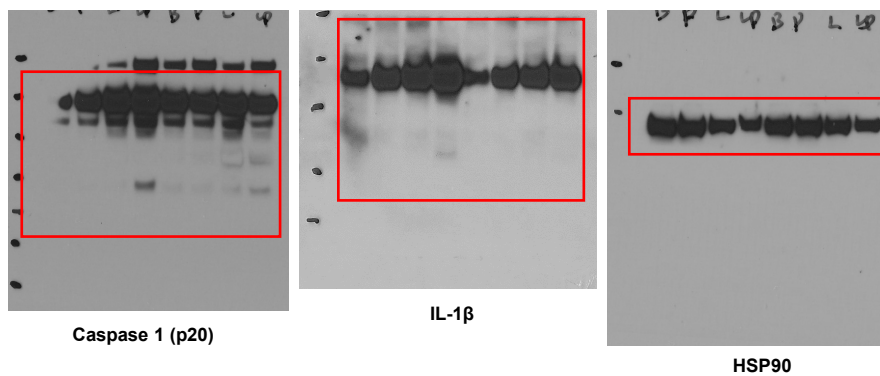

Supplement: Figure 2—source data 2. [file elife-87561-fig2-data2.pdf]

**Figure. 3E**

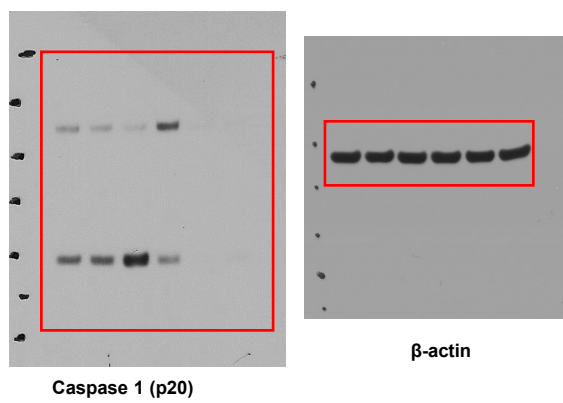

Supplement: Figure 3—source data 2. [file elife-87561-fig3-data2.pdf]

Figure. 6E

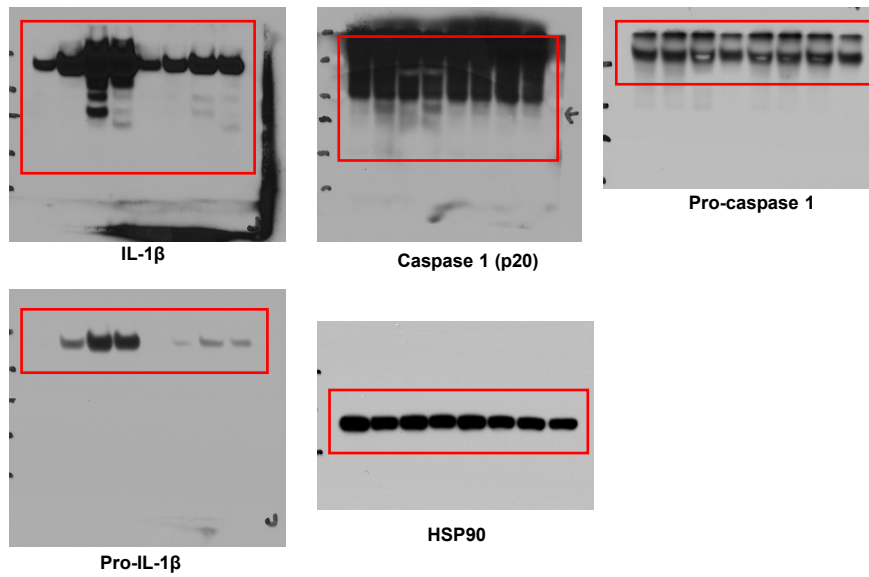

Figure. 6H

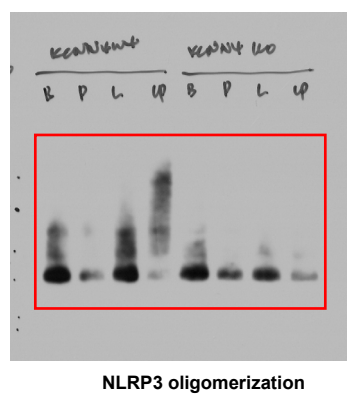

Figure. 6I

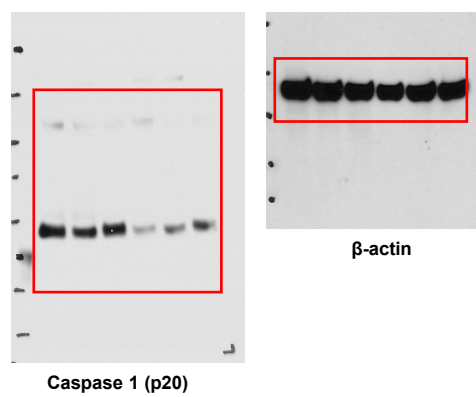

Supplement: Figure 6—source data 2. [file elife-87561-fig6-data2.pdf]

Figure. 7A

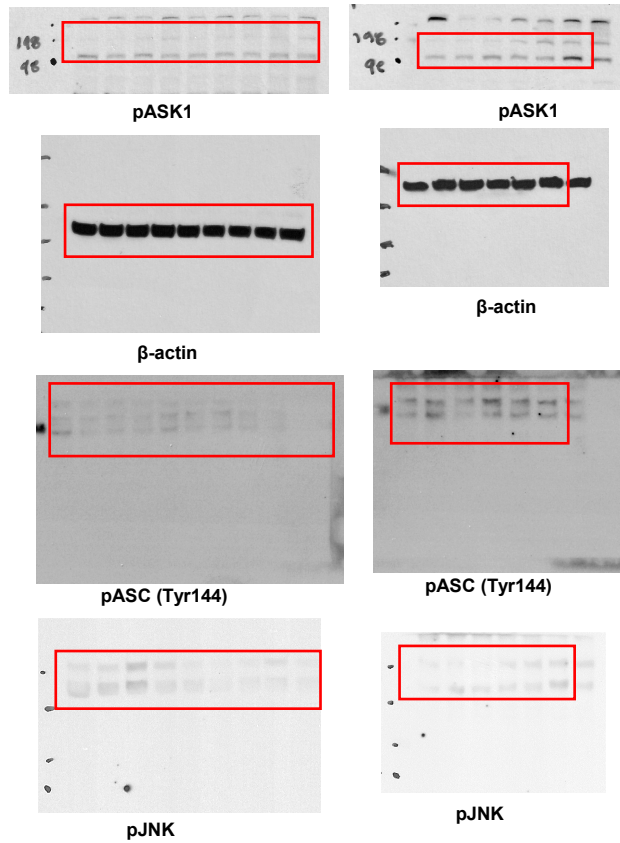

Figure. 7B

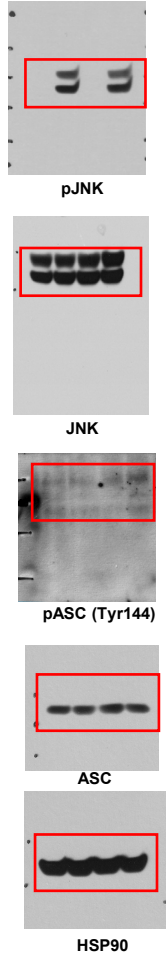

Figure. 7C

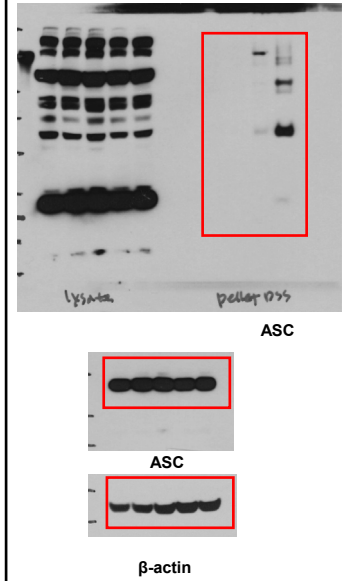

Figure. 7D

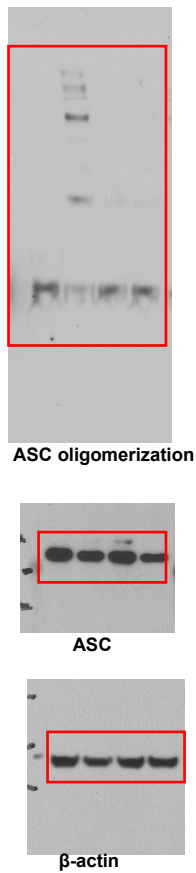

Figure. 7F

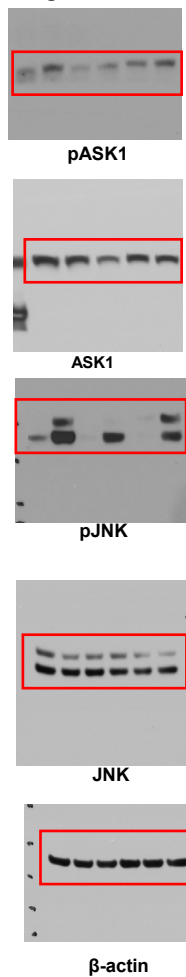

Figure. 7G

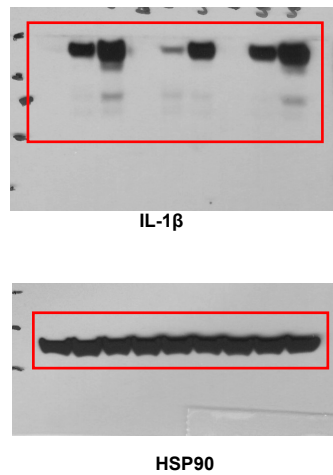

Supplement: Figure 7—source data 2. [file elife-87561-fig7-data2.pdf]
