# Supplementary material for: ER-to-lysosome Ca2+ refilling followed by K+ efflux-coupled store-operated Ca2+ entry in inflammasome activation and metabolic inflammation
Source: eLife. 2024 Jul 2;12:RP87561. doi: 10.7554/eLife.87561 (PMC11219040; doi:10.7554/eLife.87561)

Figure 7–figure supplement 1B upper

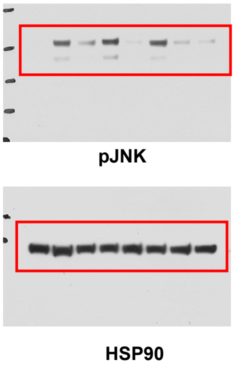

Figure 7–figure supplement 1B lower

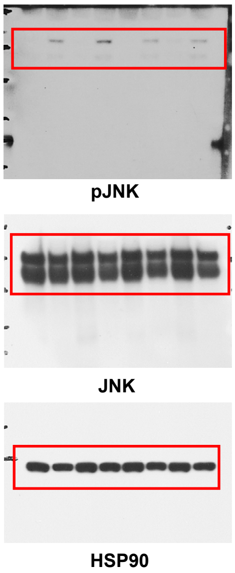

Figure 7–figure supplement 1C

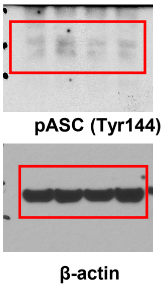

Figure 7–figure supplement 1E

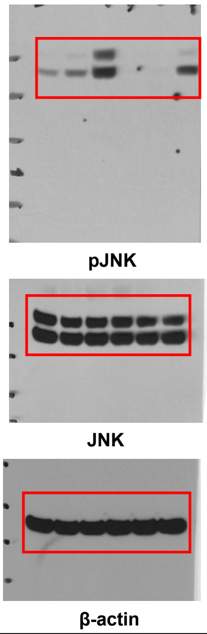

Supplement: Figure 7—figure supplement 1—source data 2. [file elife-87561-fig7-figsupp1-data2.pdf]
